# The effects of foot orthoses on radiological parameters and pain in children with flexible flat feet: a systematic review and meta-analysis

**DOI:** 10.3389/fped.2024.1388248

**Published:** 2024-08-02

**Authors:** Chao Liu, HongHao Zhang, JianPing Li, ShiJia Li, GuQiang Li, XiangZhan Jiang

**Affiliations:** Department of Special Education and Rehabilitation, Binzhou Medical University, Yantai, Shandong, China

**Keywords:** flexible flatfoot, foot orthoses, radiological parameters, pain management, meta-analysis

## Abstract

**Objective:**

This study aimed to investigate the impact of foot orthoses on foot radiological parameters and pain in children diagnosed with flexible flatfoot.

**Methods:**

A comprehensive search was conducted across several databases, including PubMed, Web of Science, EMBASE, Cochrane Library, and EBSCO, covering publications from the inception of each database up to 8 June 2024. The study focused on randomized controlled trials investigating the use of foot orthoses for treating flexible flat feet in children. Four researchers independently reviewed the identified literature, extracted relevant data, assessed the quality of the studies, and performed statistical analyses using RevMan 5.4 software.

**Results:**

Six studies involving 297 participants were included. The methodological quality of the included literature ranged from moderate to high. Radiological parameters of the foot improved significantly in older children with flexible flat feet following foot orthotic intervention compared to controls, particularly in the lateral talar-first metatarsal angle [mean difference (MD) = −2.76, 95% confidence interval (95% CI) −4.30 to −1.21, *p* = 0.0005], lateral talo-heel angle (MD = −5.14, 95% CI −7.76 to −2.52, *p* = 0.0001) and calcaneal pitch angle (MD = 1.79, 95% CI 0.88–2.69, *p* = 0.0001). These differences were statistically significant. Additionally, foot orthoses significantly improved the ankle internal rotation angle and reduced foot pain in children with symptomatic flexible flatfoot (MD = −2.51, 95% CI −4.94 to −0.07, *p* = 0.04).

**Conclusion:**

The use of foot orthoses positively impacts the improvement of radiological parameters of the foot and reduces pain in older children with flexible flat feet. However, in younger children with flexible flat feet, the improvement from foot orthoses was not significant, likely due to challenges in radiological measurements caused by the underdevelopment of the ossification centers in the foot. Further studies are needed. Consequently, the results of this meta-analysis support the implementation of an early intervention strategy using foot orthoses for the management of symptomatic flat feet in older children.

**Systematic Review Registration:**

https://www.crd.york.ac.uk/, PROSPERO [CRD42023441229].

## 1 Introduction

The foot is a crucial support and load-transmitting structure in the human body, and a healthy foot is essential for exercise and walking (1). Flat feet (FF), commonly known as flat foot syndrome, represent a musculoskeletal issue resulting from dysfunction of the foot's support structures (1, 2). This condition is characterized by the collapse or loss of the medial arch, along with displacement of the talus head and rotation of the heel bone (2, 3). Flat feet can be categorized into rigid and flexible types (4). In flexible flatfeet, the arch is present during non-weight-bearing but significantly depresses during weight-bearing (3). Rigid flat feet, which are less common, exhibit reduced arches during both weight-bearing and non-weight-bearing and often require surgical correction (5–7).

Flexible flatfoot is highly prevalent in children, with approximately 90% of children under two years of age affected by this condition. Most develop their arches rapidly between the ages of 2 and 6 years, completing them by around 10 years of age (5, 8, 9). However, some children experience abnormal growth due to structural foot abnormalities (5), and about 15% of adolescents aged ten years and older still suffer from flat feet (10). Flexible flat feet can be further classified into pathological and physiological types (11). Most cases of flexible flat feet are physiological and do not necessitate treatment (12). Despite the high prevalence of this condition in children, the majority remain asymptomatic. Research indicates that only a tiny fraction of children report pain or discomfort after physical activity, particularly in the arch region (13). These occurrences of pain are relatively rare, and in most instances, children's arches will naturally develop with age, leading to the resolution of symptoms (4).

However, if flexible flat feet are accompanied by functional impairments, they may progress to a pathological state (11). Children with pathological flat feet exhibit a significant reduction in medial arch height and impaired plantar load distribution, leading to increased stress on the foot, ankle, and knee joints and compensatory internal rotation of the hip (14). The body compensates for the abnormal posture by increasing lumbar lordosis and thoracic kyphosis (15). Prolonged exposure to an atypical biomechanical environment can lead to symptoms in children with flat feet, including foot pain during weight-bearing, easy fatigue when walking, and medial instability of the foot (16, 17). If left untreated, symptoms may worsen, resulting in secondary deformities such as stiff bunions and poor pelvic alignment (18), which can seriously affect children's health and quality of life (19).

Foot orthoses are a conservative treatment for symptomatic flat feet (20, 21). Using foot orthoses can effectively support the foot arch, inhibit talo-heel joint valgus (21), and align the foot-ankle complex closer to the normal physiological position (22, 23). Studies have shown that foot orthoses can improve the radiological parameters of the foot (18), particularly by altering the relationships among the talo-lateral-first metatarsal angle, talo-heel lateral angle, and calcaneal pitch angle. This improves the hindfoot force line and reduces pain caused by weight-bearing talo-subtalar joint subluxation (24). Additionally, foot orthoses improve lower limb joint moments, increase walking support time, reduce maximum foot pronation angle and internal tibial rotation, and enhance stride length and posture (10, 25).

Although foot orthoses have demonstrated significant clinical efficacy in reducing foot pain and lower limb symptoms (24, 25), their long-term effectiveness remains controversial. Many studies lack control groups and direct evidence supporting the significant improvement of foot radiological parameters in children with flexible flat feet through foot orthoses (26). Currently, there is no universally accepted treatment for flexible flatfoot, and studies explicitly addressing foot orthotic interventions in children are limited (20).

Radiological parameters and pain relief are crucial indicators in assessing the efficacy of foot orthoses. Radiological parameters of the foot, such as the calcaneal pitch angle, lateral talar-first metatarsal angle, and lateral talar-heel angle, can objectively evaluate the degree of arch collapse and the alignment of foot bones, thus accurately reflecting the anatomical impact of treatment (4). Pain relief is a direct clinical manifestation of treatment efficacy and is closely linked to the patient's quality of life (27). Therefore, this paper aims to systematically assess the effectiveness of foot orthoses in improving foot radiological parameters and alleviating pain in children with flexible flat feet by conducting a meta-analysis of relevant randomized controlled trials (RCT). This approach will provide a more reliable scientific basis for clinical treatment and promote the optimization and standardization of treatments for children with flexible flat feet. Through a comprehensive literature review and meta-analysis, this study seeks to fill gaps in current research and clarify the role of foot orthoses in treating flexible flatfoot, particularly their effectiveness in structural improvement of the foot and ankle and pain relief, thereby guiding clinical practice.

## 2 Materials and methods

This study adhered strictly to the Preferred Reporting Items for Systematic Reviews and Meta-Analyses (PRISMA) statement guidelines. It was registered with the International Prospective Register of Systematic Reviews (PROSPERO) under CRD42023441229.

### 2.1 Inclusion and exclusion criteria

Inclusion criteria:

(1) Study subjects were children clinically diagnosed with flexible flat feet using objective criteria such as radiological measurements of the foot, plantar pressure test, and footprint method.
(2) Study subjects were aged 2–15 years, with no restrictions on gender, nationality, or duration of treatment.
(3) The experimental group was treated with foot orthoses, while the control group received no intervention or prosthetic insoles without corrective effects.
(4) The study design was a randomized controlled trial.

Exclusion criteria:

(1) Children with rigid flat feet.
(2) Non-clinical randomized controlled trials.
(3) Studies where the intervention modality and outcome metrics did not meet the trial requirements.

### 2.2 Interventions

#### 2.2.1 Control group

2 mm thick flat insoles or no intervention.

#### 2.2.2 Experimental group

Foot orthotics.

### 2.3 Outcome measures

#### 2.3.1 Primary indicators

Lateral talo-first metatarsal angle, lateral talo-heel angle, and calcaneal pitch angle.

#### 2.3.2 Secondary indicators

Pain, foot external rotation angle.

### 2.4 Literature search strategy

To conduct the systematic review and meta-analysis, a detailed literature search strategy was developed to ensure the acquisition of relevant and high-quality research data. The specific steps of the search strategy are as follows:

#### 2.4.1 Identify databases

Multiple relevant databases were selected to ensure a comprehensive and rigorous search. The databases included in the search are PubMed, Web of Science, Embase, Cochrane Library, and EBSCO.

#### 2.4.2 Search terms

To conduct a thorough and systematic review, we performed a comprehensive literature search utilizing Medical Subject Headings (MeSH) and keywords structured around the PICO (Population, Intervention, Comparison, Outcome) framework. This approach allowed us to identify and include studies most pertinent to our research questions. For full transparency and reproducibility, the detailed search strategy—including specific search terms, databases utilized, and search strings—is documented in the Supplementary File.

#### 2.4.3 Combined search form (using the EBSCO database as an example)

(Flatfoot OR Talipes Valgus OR Valgus, Talipes OR Splayfoot OR Flat Foot OR Foot, Flat OR Pes Planus OR Flat Feet OR Feet, Flat OR Flatfeet OR Vertical Talus OR Talus, Vertical OR Rigid Flatfoot OR Flatfoot, Rigid OR Convex Foot OR Foot, Convex OR Convex Pes Valgus OR Pes Valgus, Convex OR Vertical Talus, Congenital OR Congenital Vertical Talus OR Talus, Congenital Vertical OR Rocker-Bottom Foot OR Foot, Rocker-Bottom OR Rocker Bottom Foot OR Pes Valgus, Congenital Convex OR Talipes Calcaneovalgus OR Calcaneovalgus, Talipes OR Flexible Flatfoot OR Flatfoot, Flexible OR Acquired Adult Flatfoot Deformity) AND (children OR Flatfoot OR Child) AND (Foot Orthoses OR Orthoses, Foot OR Foot Orthosis OR Orthosis, Foot OR Foot Orthotic Devices OR Device, Foot Orthotic OR Devices, Foot Orthotic OR Foot Orthotic Device OR Orthotic Device, Foot OR Orthotic Devices, Foot OR Foot Arch Supports OR Arch Support, Foot OR Arch Supports, Foot OR Foot Arch Support OR Support, Foot Arch OR Supports, Foot Arch OR Orthotic Shoe Inserts OR Insert, Orthotic Shoe OR Inserts, Orthotic Shoe OR Orthotic Shoe Insert OR Shoe Insert, Orthotic OR Shoe Inserts, Orthotic OR Orthotic Insoles OR Insole, Orthotic OR Insoles, Orthotic OR Orthotic Insole) AND (control OR comparison OR treatment OR intervention OR Control Groups OR Comparative Study) AND (radiographic parameters OR pain relief OR foot pain OR improvement OR Pain Measurement OR Pain Relief).

#### 2.4.4 Scope of search

Articles published up to 8 June 2024 were retrieved. The literature types included journals and dissertations. Additional searches were conducted to identify references cited in the included literature and relevant reviews to minimize the risk of missing relevant literature.

#### 2.4.5 Literature screening

Three authors (LC, ZHH, and LJP) collaboratively performed a comprehensive literature search across five databases: PubMed, Embase, Cochrane Library, EBSCO, and Web of Science. Each author independently screened the identified articles using predefined inclusion and exclusion criteria. Discrepancies in the selection process were resolved through discussion and consensus among the three authors. Additionally, each included article underwent a double-checking process to ensure accurate and consistent data extraction. Another author (LSJ) used Note Express to eliminate duplicate records. Two authors (LJP and LSJ) independently assessed the titles and abstracts of each article to identify literature that initially met the inclusion criteria. Subsequently, two authors (LC and ZHH) read the full texts of the included articles to eliminate those that did not meet the study requirements, following the exclusion criteria. In cases of disagreement among the four authors on whether an article should be included, the matter was referred to senior scholars (JXZ and LGQ) for further review to decide on inclusion.

#### 2.4.6 Literature extraction

After reviewing the complete literature, two authors (LC and ZHH) independently extracted and cross-checked relevant data. The extracted data included:

(1) Basic information (author, country, year of publication, sample size, age, gender);
(2) Intervention mode and duration;
(3) Outcome indicators.

#### 2.4.7 Assessment of literature quality

The quality of the primary literature included in this paper was assessed using the Cochrane Collaboration's risk of bias assessment tool (28). The assessment, conducted by author LC, included evaluation of:

(1) Random allocation methods;
(2) Allocation concealment;
(3) Blinding of researchers and subjects;
(4) Blinding in the assessment of study endpoints;
(5) Completeness of endpoint data;
(6) Selective reporting of endpoints;
(7) Other sources of bias.

#### 2.4.8 Statistical analysis

Statistical analyses were performed using RevMan 5.4 software for meta-analysis to assess the effectiveness of foot orthoses in improving radiological parameters and pain in children with flexible flat feet. For continuous variables, the weighted mean difference (WMD) or standardized mean difference (SMD) was used, and the mean difference and 95% confidence interval (CI) were extracted for each outcome indicator. Heterogeneity was analyzed using the χ^2^ test (*α *= 0.1); if *I*^2^ < 50%, the study was considered homogeneous, and a fixed-effects model was used to calculate the composite effect size. If *I*^2^ ≥ 50%, the study was considered heterogeneous and analyzed using a random-effects model to determine the source of heterogeneity, enabling subgroup analyses or sensitivity analyses. The significance level was set at *α* = 0.05. If there were more than ten papers for the primary outcome, funnel plots were drawn to detect publication bias.

## 3 Results

### 3.1 Results of literature screening

**Figure 1:**
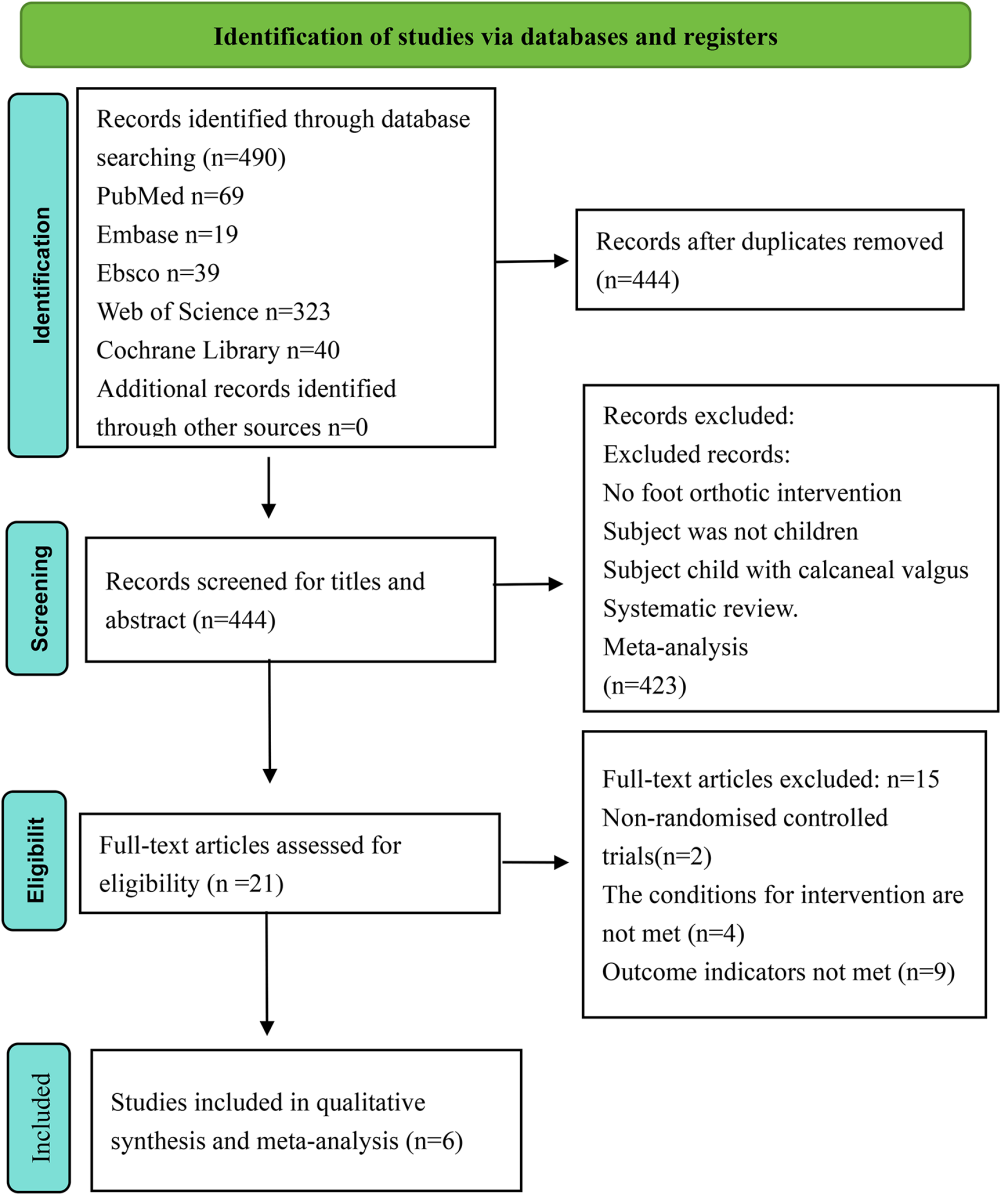
Literature screening flowchart.

According to our search strategy, we obtained 490 relevant articles from various databases: 69 from PubMed, 19 from Embase, 39 from Ebsco, 323 from Web of Science, and 40 from Cochrane Library, with no additional records identified through other sources. After removing 46 duplicates, we reviewed the titles and abstracts of the remaining 444 articles. Of these, 423 articles were excluded for the following reasons: no foot orthotic intervention, subjects were not children, subjects were children with calcaneal valgus, systematic reviews, or meta-analyses. We then reviewed the full texts of the remaining articles, excluding two that were not randomized controlled trials, four that used foot orthoses in both experimental and control groups, and nine that did not measure radiological parameters of the foot and ankle region or pain scores. Six eligible papers involving 297 children with flexible flat feet were included. The flow chart for the literature screening process is detailed in Figure 1.

Six studies were included in this meta-analysis, involving 297 children with flexible flat feet, comprising 189 males and 108 females. All studies were conducted in Asia, with sample sizes ranging from 29 to 81 cases, and the age range of the children was from 2 to 15 years. One study included only male subjects (29). Various types of foot orthoses were used for intervention correction across the six studies. Regarding control group settings, four studies did not use any intervention (24, 30–32), while two studies used 2-mm insoles as a control (29, 33). The duration of the intervention varied, ranging from a minimum of 12 weeks to a maximum of 34.6 ± 10.9 months. Different methods were used to assess the effects of the intervention in the six studies. Three studies evaluated radiological parameters of the foot and ankle region, including changes in the lateral talar-first metatarsal angle, lateral talo-heel angle, and calcaneal pitch angle (24, 30, 31); two studies used pain scores (32, 33); and one study assessed the angle of internal ankle rotation (29). These studies provide extensive data for analyzing the effects of correction in children with flexible flat feet through various assessment methods and interventions. Data related to study characteristics are detailed in Table 1.

**Table 1:** The general characteristics of the studies included.

Researcher	Country	Age (T/C)	Sex (male/female)	Number of subjects (T/C)	Intervention time	Type of intervention (T/C)	Outcome indicator	Main findings
Sinha et al. (24)	South Korea	8.25 ± 4.25/8.33 ± 4.42	48/33	55/26	4 months	Customized orthopedic insoles/No intervention	ΔLateral talo-first metatarsal angle ΔLateral talocalcaneal angle ΔCalcaneal pitch angle	Significant improvement in AOFAS scores and most foot angles in orthosis group, only partial improvement in control group.
Kanatlı et al. (31)	Istanbul	3.37 ± 1.04	33/12	21/24	34.6 ± 10.9 months	Orthopedic shoes/No intervention	ΔLateral talo-first metatarsal angle ΔLateral talocalcaneal angle ΔCalcaneal pitch angle	No significant effect of corrective shoes on foot arch development, should be limited to selected cases.
Lee et al. (30)	South Korea	9.16 ± 3.53/7.73 ± 2.37	20/9	16/13	15.44 ± 5.49 months/15.62 ± 6.78 months	Pressurized 3D printed insoles /No intervention	ΔLateral talo-first metatarsal angle ΔLateral talocalcaneal angle ΔCalcaneal pitch angle	Some effect on hindfoot bony alignment, but no significant change in midfoot pressure.
Hsieh et al. (33)	Taiwan	6.9 ± 0.6/6.2 ± 0.4	28/24	26/26	12 weeks	Customized arch support insoles/2 mm thick flat insoles	Pain	Significant improvement in pain/comfort, physical health, stair ascent time, upper extremity and physical function, transfer and basic mobility.
Asgaonkar and Kadam (32)	India	9.40 ± 2.66/9.30 ± 2.44	30/30	30/30	6 months	Customized orthopedic insoles /No intervention	Pain	Significant improvement in pain and PCI, no significant change in gait parameters.
Jafarnezhadgero et al. (29)	Iranian	10.5 ± 1.4/10.4 ± 1.5	30/0	15/15	4 months	Medial arch support foot orthoses/2 mm thick flat insoles	Ankle internal rotation	Significant improvement in lower limb alignment and ground reaction forces.

T is the experimental group and C is the control group; Δ, delta.

### 3.2 Quality assessment of the included literature

**Figure 2:**
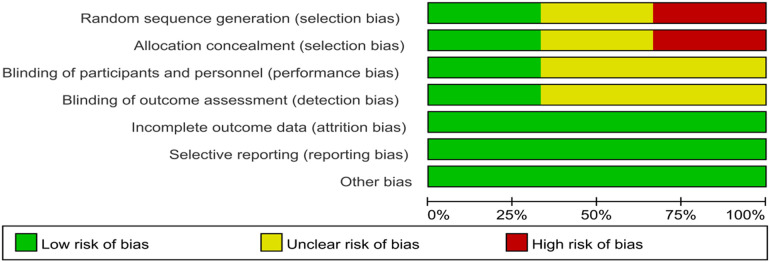
Risk of bias graph.

**Figure 3:**
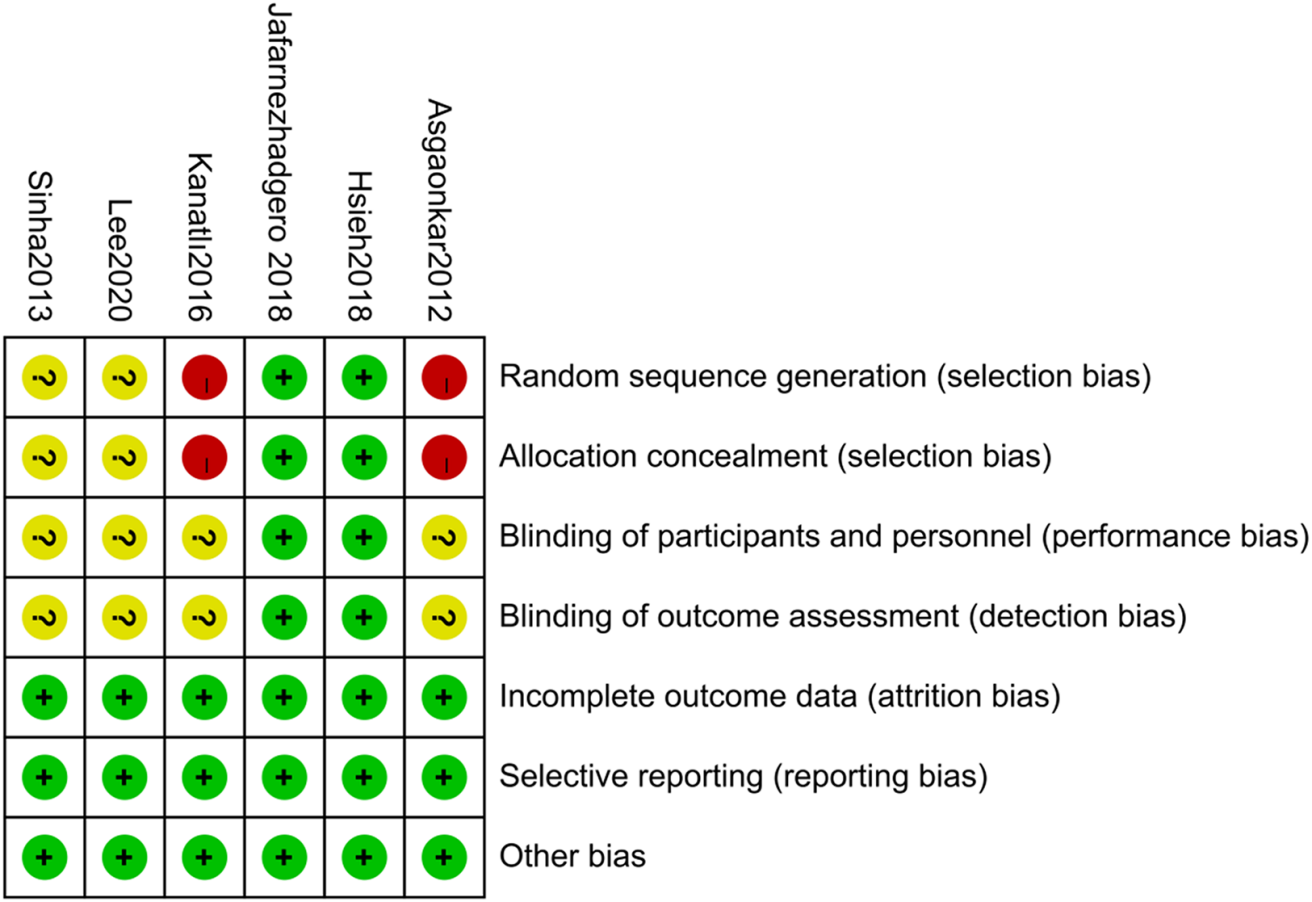
Risk of bias summary.

Based on the Cochrane Risk of Bias Assessment Scale, we conducted a detailed assessment of the six included studies with the following results:

Two studies introduced a randomized sequence approach and were classified as low risk (29, 33); one study was grouped based on the date of examination and was classified as high risk (31); one study was grouped based on gender and age and was classified as high risk (32), and the remaining two studies did not specify their grouping method (24, 30). Two studies described allocation concealment (29, 33); one study was classified as high risk based on the date of examination (31); one study was classified as high risk based on gender and age (32), and the remaining two studies did not specify their allocation method (24, 30). Two studies used blinding (29, 33), while the other four studies did not use blinding (24, 30–32), although outcome judgments and measurements were not likely affected. All six studies reported complete data without selective reporting, and no other sources of bias were identified. The results of the quality assessment are shown in Figures 2, 3.

### 3.3 Meta-analysis

#### 3.3.1 Effect of foot orthoses on radiological parameters of the foot in children with flexible flat feet

Three studies were included in the meta-analysis (24, 30, 31). The results showed that, compared with the control group, foot orthoses significantly improved radiological parameters of the foot and ankle region in older children with flexible flat feet. Specifically, foot orthoses changed the angular relationship between the lateral talar-first metatarsal angle, the lateral talo-heel angle, and the calcaneal pitch angle, improving the hindfoot line of force and relieving foot discomfort. In younger children with flat feet, the improvement effect of orthotics is not apparent due to the underdevelopment of the ossification center of the foot and the difficulty of radiological measurements. Further studies are needed. The specific meta-analysis results are as follows:

(1) Lateral Talar-First Metatarsal Angle

**Figure 4:**
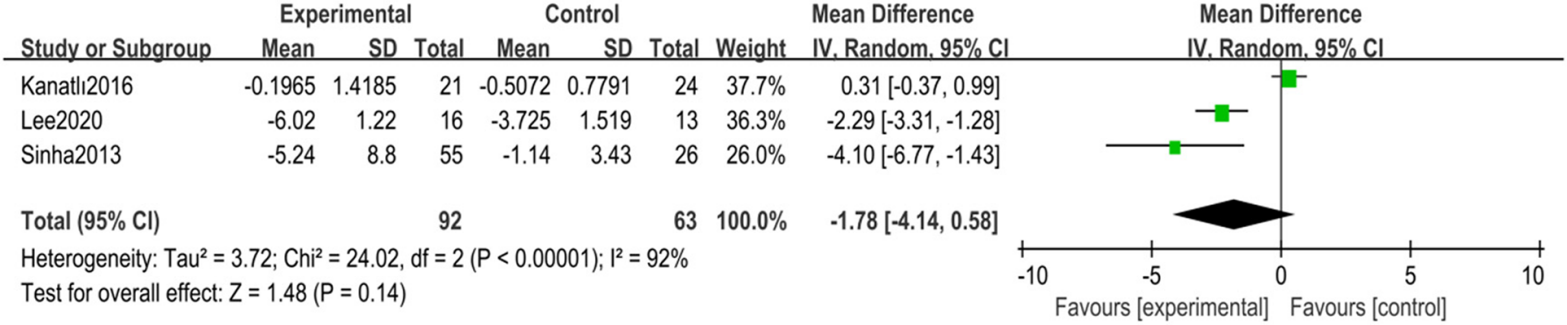
Forest plot of lateral talar-first metatarsal angle in children with flexible flat feet.

A total of three randomized controlled trials (24, 30, 31) with 155 children with flexible flatfoot were included. Analyses were performed using a random-effects model, which showed high heterogeneity (*I*^2^ = 92%, *p* < 0.00001), indicating significant differences between the study results. Despite the pooled analysis, the overall effect was insignificant (MD = −1.78, 95% CI −4.14–0.58, *p* = 0.14) (Figure 4).

**Figure 5:**
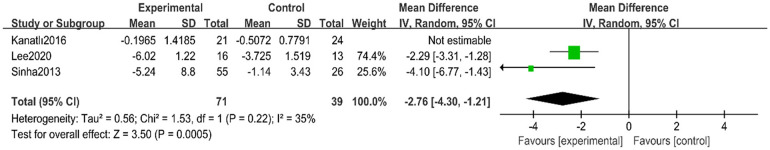
Forest plot of lateral talar-first metatarsal angle after heterogeneity analysis.

Sensitivity analyses were conducted to explore sources of heterogeneity. After removing one study (31), heterogeneity was significantly reduced to moderate levels (*I*^2^ = 35%, *p* = 0.22), and the overall effect estimate became statistically significant (MD = −2.76, 95% CI −4.30 to −1.21, *p* = 0.0005) (Figure 5).

(2) Lateral Talo-Heel Angle

**Figure 6:**
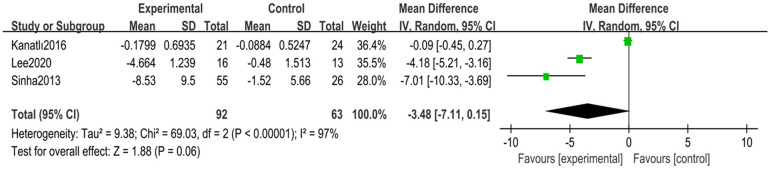
Forest plot of lateral talo-heel angle in children with flexible flat feet.

A total of three randomized controlled trials (24, 30, 31) with 155 children with flexible flatfoot were included. Analyses were performed using a random-effects model, which showed high heterogeneity (*I*^2^ = 97%, *p* < 0.00001). Despite the significant heterogeneity, the overall effect was statistically significant (MD = −3.48, 95% CI −7.11–0.15, *p* = 0.06) (Figure 6).

**Figure 7:**
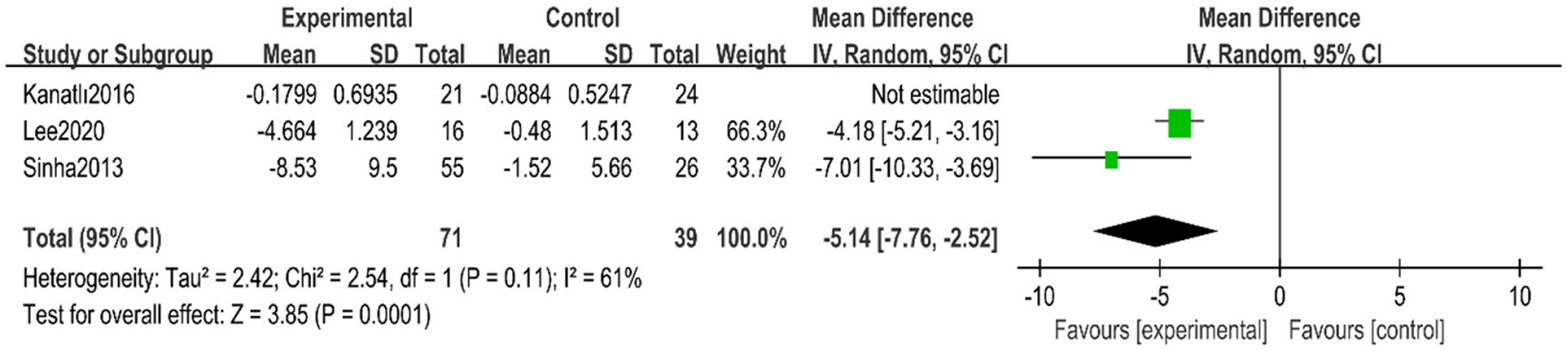
Forest plot of lateral talar-first metatarsal angle after heterogeneity analysis.

Sensitivity analyses were conducted to explore sources of heterogeneity. After removing one study (31), heterogeneity was significantly reduced (*I*^2^ = 61%, *p* = 0.11), and the overall effect result remained statistically significant (MD = −5.14, 95% CI −7.76 to −2.52, *p* = 0.0001) (Figure 7).

(3) Calcaneal Pitch Angle

**Figure 8:**
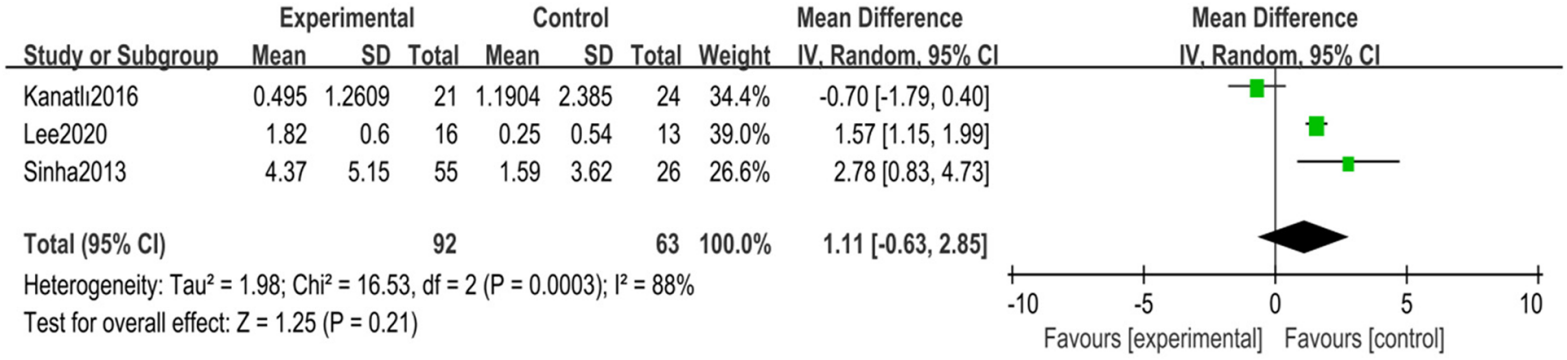
Forest plot of calcaneal pitch angle in children with flexible flat feet.

A total of three randomized controlled trials (24, 30, 31) with 155 children with flexible flatfoot were included. Analyses were performed using a random-effects model, which showed high heterogeneity (*I*^2^ = 88%, *p* = 0.0003), indicating significant differences between the study results. Despite the pooled analysis, the overall effect was insignificant (MD = 1.11, 95% CI −0.63–2.85, *p* = 0.21) (Figure 8).

**Figure 9:**
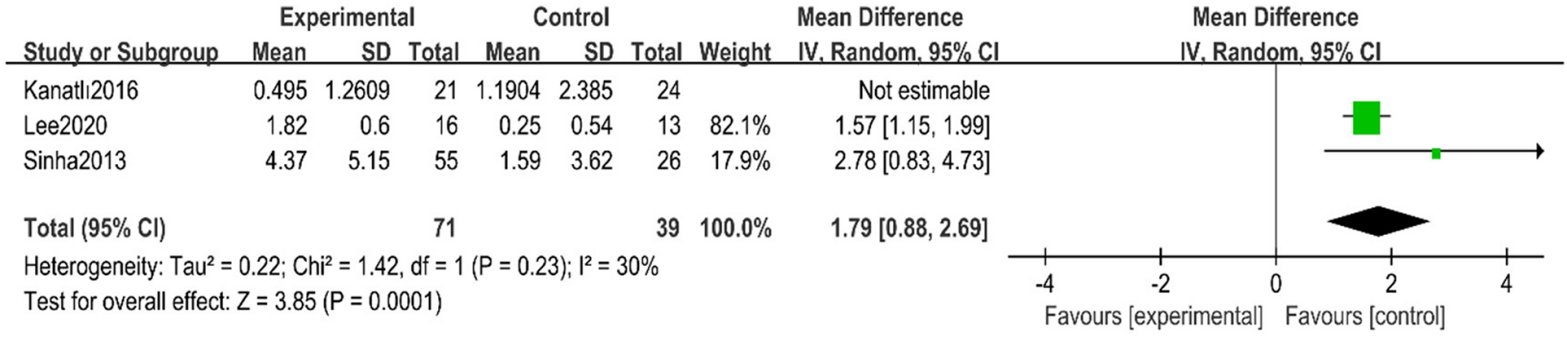
Forest plot of calcaneal pitch angle after heterogeneity analysis.

Sensitivity analyses were conducted to explore sources of heterogeneity. After removing one study (31), heterogeneity was significantly reduced to moderate levels (*I*^2^ = 30%, *p* = 0.23), and the overall effect estimate became statistically significant (MD = 1.79, 95% CI 0.88–2.69, *p* = 0.0001) (Figure 9).

#### 3.3.2 Effect of foot orthoses on pain in the ankle region in children with flexible flat feet

**Figure 10:**
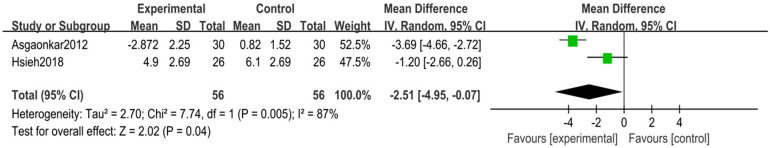
Forest plot of pain in children with flexible flat feet.

The meta-analysis included two studies (32, 33), which showed that foot orthoses reduced foot and ankle pain by providing additional arch support, reducing stress caused by arch collapse, and improving foot biomechanics compared to the control group. The specific meta-analysis results are as follows:

Two randomized controlled trials totaling 112 children with flexible flat feet were included. Analyses were performed using a random-effects model, which showed high heterogeneity (*I*^2^ = 87%, *p* = 0.005). Despite the significant heterogeneity, the overall effect was statistically significant (MD = −2.51, 95% CI −4.94 to −0.07, *p* = 0.04). Heterogeneity was not further analyzed due to the few included studies (Figure 10).

#### 3.3.3 Effect of foot orthoses on ankle internal rotation in children with flexible flat feet

One study was included in the analysis (29), which showed that foot orthoses effectively reduced the degree of internal rotation of the foot compared to the control group by providing arch support and improving the distribution of forces in the foot. Additionally, applying foot orthoses affected the vertical ground reaction force during locomotion, improving gait and balance. A meta-analysis was not performed due to the limited number of included studies.

#### 3.3.4 Heterogeneity analysis

This study showed significant heterogeneity in the preliminary meta-analysis, which may be attributed to several factors.

##### 3.3.4.1 Age differences

Three studies were included in the analysis of radiological parameters (24, 30, 31). In one study (31), the mean age of the children with flexible flat feet was four years, significantly lower than the mean age in the other two studies (24, 30). Age differences may lead to variations in foot development and response to foot orthotic interventions, significantly affecting study outcomes. Differences in foot structure and function between younger and slightly older children can impact the efficacy of the intervention and the measurement of radiological parameters. Up to the age of four, children may face difficulties in radiological measurements due to the underdevelopment of the ossification center of the foot, affecting experimental results (24, 30).

##### 3.3.4.2 Duration of intervention

Three studies were included in the analysis of radiological parameters (24, 30, 31). In one study (31), the duration of intervention for children with flexible flat feet was 36 months, significantly longer than in the other two studies (24, 30). Longer intervention durations may lead to more pronounced outcome changes but can also introduce more variables and uncertainty. Different intervention durations may affect the foot's adaptation process, impacting the outcome differently.

Fewer than ten randomized controlled trials were included in this study's outcome metrics, so funnel plots were not used to test for risk of bias.

## 4 Discussion

This study aimed to investigate the effect of foot orthoses on foot radiological parameters and pain in children with flexible flat feet. The results showed that foot orthoses significantly improved the radiological parameters of the foot in older children, particularly in terms of the lateral talar-first metatarsal angle, lateral talo-heel angle, and calcaneal pitch angle. These improvements included adjusting the ankle's internal rotation angle and optimizing the hindfoot line of force to achieve correction. In younger children, it has not been possible to determine the improvement effect of foot orthoses due to the complexity of the foot tissue and the fact that the center of ossification is not apparent until the age of four years, making radiological measurements more complex (30). In addition, this study found that children with symptomatic flat feet who used foot orthoses experienced significantly less pain when walking, improved gait and posture, and enhanced comfort and mobility in daily life.

### 4.1 Effect of foot orthoses on radiological parameters of the foot in children with flexible flat feet

Radiological parameters of the foot can effectively reveal the impact of foot orthoses on foot structure. The main radiological parameters in this study included the lateral talar-first metatarsal angle, lateral talo-heel angle, and calcaneal pitch angle. The lateral talar-first metatarsal angle reflects the alignment of the midfoot with the forefoot in the horizontal plane and is an essential measure of talar inclination (34). The lateral talo-heel angle is a crucial radiological parameter in the evaluation of flatfoot, indirectly reflecting arch height and hindfoot alignment (9). The calcaneal pitch angle, on the other hand, reflects the alignment of the hindfoot with the forefoot in the sagittal plane, predicting the risk of developing flatfoot symptoms (35), and is one of the best indicators for the diagnosis of flatfoot due to its high sensitivity and specificity (36).

Children with flexible flat feet usually present with heel valgus and an increased talar inclination angle, which leads to internal rotation of the hindfoot and a low arch, altering the dynamics of the foot chain and potentially overstressing the subtalar and mid-tarsal joints, which can lead to ankle injuries (24, 29). The present meta-analysis found that foot orthoses effectively corrected older children and significantly improved foot radiological parameters. By supporting the medial arch, foot orthoses alter the alignment between the talus and the heel bone (30), adjusting the relationship between the lateral talar-first metatarsal angle, the lateral talo-heel angle, and the calcaneal pitch angle, thereby facilitating hindfoot alignment and arch restoration (24). Additionally, foot orthoses correct and improve walking gait and posture by supporting the medial arch and enhancing the ankle joint angle and moment (37).

### 4.2 Effect of foot orthoses on pain in the foot and ankle region in children with flexible flat feet

The meta-analysis revealed that foot orthoses have a significant impact on pain management in children with flexible flat feet. In these children, the medial arch height is notably reduced, causing an uneven distribution of plantar pressure and mechanical instability of the foot (38). To preserve the arch and restrict over-rotation for balance, the intrinsic foot muscles and tibialis posterior muscles must be highly active (38). This heightened activity predisposes these muscles to overuse injuries and entrapment pain, particularly due to tibialis posterior myofascial pain syndrome (39).

Foot orthoses address these issues by redistributing plantar pressure through medial arch support, increasing peak pressure on the hallux and metatarsals, and enhancing the midfoot contact area (40, 41). Specifically, foot orthoses modulate the activity of the anterior tibialis, posterior tibialis, and peroneal muscles, thereby strengthening the midfoot region to enhance arch stability and provide superior structural support to the medial longitudinal arch (42, 43). They also reduce the amplitude and duration of abnormal pronation during the support phase, decrease plantar ligament stress, and alleviate plantar pain (44). Studies have demonstrated that by adjusting the medial arch, orthoses can significantly alleviate pain in children with flexible flat feet. They modify radiological parameters and improve hindfoot alignment during weight-bearing, reducing subtalar joint inclination and talonavicular subluxation (24, 32). Additionally, orthoses decrease the workload on foot muscles, delay muscle fatigue, and enhance foot balance and postural control (38).

However, prolonged use of foot orthoses can diminish the size and activity of intrinsic muscles, resulting in muscle atrophy. Consequently, integrating foot orthoses with appropriate muscle-strengthening exercises is advisable to preserve and enhance intrinsic muscle activity, thereby ensuring sustained support and stability for the foot (43).

### 4.3 Limitations

Although this study evaluated the effectiveness of foot orthoses in improving radiological parameters and pain in the feet of children with flexible flat feet and made some meaningful findings, there are limitations. First, the small sample size of the included studies may have affected the validity of the meta-analysis results and the accuracy of the conclusions. The limited sample size resulted in restricted stability and representativeness of the results. Therefore, future studies need to include larger samples to improve the reliability and applicability of the findings. Secondly, the present study found that orthotics improved foot radiological parameters in older children with flat feet, but the effect was insignificant in younger children. This may be related to age-related foot developmental stages and orthotic adaptations. As the foot is not fully developed in younger children, the effect of orthoses at this specific implementation stage may need to be clarified, and further research is needed to explore this issue.

## 5 Conclusion

This meta-analysis demonstrated that foot orthoses are effective in older children with flexible flat feet. Specifically, orthoses significantly improved radiological parameters of the foot (e.g., lateral talar-first metatarsal angle, lateral talo-heel angle, and calcaneal pitch angle), promoting arch restoration and rearfoot alignment. Additionally, the orthoses effectively reduced foot pain, improved gait and posture, and enhanced comfort and mobility in daily life in these children. However, the small sample size of this study may affect the representativeness and stability of the results. Therefore, more high-quality studies and trials with larger sample sizes are needed to validate these findings and further explore the applicability and efficacy of orthoses in children of different ages. In conclusion, foot orthoses have significant potential in treating children with flexible flat feet and provide clinical solid support. Future studies should optimize the design and use of orthoses to improve their therapeutic efficacy further and ultimately enhance the quality of life of children with flexible flat feet.

## Data Availability

The original contributions presented in the study are included in the article/Supplementary Material, further inquiries can be directed to the corresponding authors.
