# Supplementary material for: The effects of foot orthoses on radiological parameters and pain in children with flexible flat feet: a systematic review and meta-analysis
Source: Front Pediatr. 2024 Aug 2;12:1388248. doi: 10.3389/fped.2024.1388248 (PMC11327157; doi:10.3389/fped.2024.1388248)
Supplement: Supplementary file 1 [file Datasheet1.docx]

**Supplementary file**

**1.Databases：**PubMed, Web of Science, Embase, Cochrane Library, and EBSCO.

**2.Search terms（Subject terms and keywords）**

Searches were conducted using Medical Subject Headings (MeSH) and keywords associated with the PICO elements.

**Population**

**MeSH Terms:** Flatfoot, Child

**Keywords:** Talipes Valgus, Valgus, Talipes, Splayfoot, Flat Foot, Foot, Flat, Pes Planus, Flat Feet, Feet, Flat, Flatfeet, Vertical Talus, Talus, Vertical, Rigid Flatfoot, Flatfoot, Rigid, Convex Foot, Convex Pes Valgus, Pes Valgus, Convex, Vertical Talus, Congenital, Congenital Vertical Talus, Talus, Congenital Vertical, Rocker-Bottom Foot, Foot, Rocker-Bottom, Rocker Bottom Foot, Pes Valgus, Congenital Convex, Talipes Calcaneovalgus, Calcaneovalgus, Talipes, Flexible Flatfoot, Flatfoot, Flexible, Acquired Adult Flatfoot Deformity, children, pediatric

**Intervention**

**MeSH Terms:** Foot Orthoses

**Keywords:** Orthoses, Foot, Foot Orthosis, Orthosis, Foot, Foot Orthotic Devices, Device, Foot Orthotic, Devices, Foot Orthotic, Foot Orthotic Device, Orthotic Device, Foot, Orthotic Devices, Foot, Foot Arch Supports, Arch Support, Foot, Arch Supports, Foot, Foot Arch Support, Support, Foot Arch, Supports, Foot Arch, Orthotic Shoe Inserts, Insert, Orthotic Shoe, Inserts, Orthotic Shoe, Orthotic Shoe Insert, Shoe Insert, Orthotic, Shoe Inserts, Orthotic, Orthotic Insoles, Insole, Orthotic, Insoles, Orthotic, Orthotic Insole

**Comparison**

**MeSH Terms:** Control Groups, Comparative Study

**Keywords:** Control, Comparison, Treatment, Intervention

**Outcome**

**MeSH Terms:** Pain Measurement, Pain Relief

**Keywords:** Radiographic Parameters, Pain Relief, Foot Pain, Improvement

**3.Search strings**

**EBSCO :** (Flatfoot OR Talipes Valgus OR Valgus, Talipes OR Splayfoot OR Flat Foot OR Foot, Flat OR Pes Planus OR Flat Feet OR Feet, Flat OR Flatfeet OR Vertical Talus OR Talus, Vertical OR Rigid Flatfoot OR Flatfoot, Rigid OR Convex Foot OR Foot, Convex OR Convex Pes Valgus OR Pes Valgus, Convex OR Vertical Talus, Congenital OR Congenital Vertical Talus OR Talus, Congenital Vertical OR Rocker-Bottom Foot OR Foot, Rocker-Bottom OR Rocker Bottom Foot OR Pes Valgus, Congenital Convex OR Talipes Calcaneovalgus OR Calcaneovalgus, Talipes OR Flexible Flatfoot OR Flatfoot, Flexible OR Acquired Adult Flatfoot Deformity) AND (children OR Flatfoot OR Child) AND (Foot Orthoses OR Orthoses, Foot OR Foot Orthosis OR Orthosis, Foot OR Foot Orthotic Devices OR Device, Foot Orthotic OR Devices, Foot Orthotic OR Foot Orthotic Device OR Orthotic Device, Foot OR Orthotic Devices, Foot OR Foot Arch Supports OR Arch Support, Foot OR Arch Supports, Foot OR Foot Arch Support OR Support, Foot Arch OR Supports, Foot Arch OR Orthotic Shoe Inserts OR Insert, Orthotic Shoe OR Inserts, Orthotic Shoe OR Orthotic Shoe Insert OR Shoe Insert, Orthotic OR Shoe Inserts, Orthotic OR Orthotic Insoles OR Insole, Orthotic OR Insoles, Orthotic OR Orthotic Insole) AND (control OR comparison OR treatment OR intervention OR Control Groups OR Comparative Study) AND (radiographic parameters OR pain relief OR foot pain OR improvement OR Pain Measurement OR Pain Relief).

**PubMed：**(("Flatfoot"[MeSH] OR "Talipes Valgus" OR "Valgus" OR "Talipes" OR "Splayfoot" OR "Flat Foot" OR "Foot, Flat" OR "Pes Planus" OR "Flat Feet" OR "Feet, Flat" OR "Flatfeet" OR "Vertical Talus" OR "Talus, Vertical" OR "Rigid Flatfoot" OR "Flatfoot, Rigid" OR "Convex Foot" OR "Convex Pes Valgus" OR "Pes Valgus, Convex" OR "Vertical Talus, Congenital" OR "Congenital Vertical Talus" OR "Talus, Congenital Vertical" OR "Rocker-Bottom Foot" OR "Foot, Rocker-Bottom" OR "Rocker Bottom Foot" OR "Pes Valgus, Congenital Convex" OR "Talipes Calcaneovalgus" OR "Calcaneovalgus, Talipes" OR "Flexible Flatfoot" OR "Flatfoot, Flexible" OR "Acquired Adult Flatfoot Deformity" OR children OR pediatric) AND ("Foot Orthoses"[MeSH] OR "Orthoses, Foot" OR "Foot Orthosis" OR "Orthosis, Foot" OR "Foot Orthotic Devices" OR "Device, Foot Orthotic" OR "Devices, Foot Orthotic" OR "Foot Orthotic Device" OR "Orthotic Device, Foot" OR "Orthotic Devices, Foot" OR "Foot Arch Supports" OR "Arch Support, Foot" OR "Arch Supports, Foot" OR "Foot Arch Support" OR "Support, Foot Arch" OR "Supports, Foot Arch" OR "Orthotic Shoe Inserts" OR "Insert, Orthotic Shoe" OR "Inserts, Orthotic Shoe" OR "Orthotic Shoe Insert" OR "Shoe Insert, Orthotic" OR "Shoe Inserts, Orthotic" OR "Orthotic Insoles" OR "Insole, Orthotic" OR "Insoles, Orthotic" OR "Orthotic Insole") AND ("Control Groups"[MeSH] OR "Comparative Study"[MeSH] OR Control OR Comparison OR Treatment OR Intervention) AND ("Pain Measurement"[MeSH] OR "Pain Relief"[MeSH] OR "Radiographic Parameters" OR "Pain Relief" OR "Foot Pain" OR Improvement))

**Web of Science：**TS=("Flatfoot" OR "Talipes Valgus" OR "Valgus" OR "Talipes" OR "Splayfoot" OR "Flat Foot" OR "Foot, Flat" OR "Pes Planus" OR "Flat Feet" OR "Feet, Flat" OR "Flatfeet" OR "Vertical Talus" OR "Talus, Vertical" OR "Rigid Flatfoot" OR "Flatfoot, Rigid" OR "Convex Foot" OR "Convex Pes Valgus" OR "Pes Valgus, Convex" OR "Vertical Talus, Congenital" OR "Congenital Vertical Talus" OR "Talus, Congenital Vertical" OR "Rocker-Bottom Foot" OR "Foot, Rocker-Bottom" OR "Rocker Bottom Foot" OR "Pes Valgus, Congenital Convex" OR "Talipes Calcaneovalgus" OR "Calcaneovalgus, Talipes" OR "Flexible Flatfoot" OR "Flatfoot, Flexible" OR "Acquired Adult Flatfoot Deformity" OR children OR pediatric) AND TS=("Foot Orthoses" OR "Orthoses, Foot" OR "Foot Orthosis" OR "Orthosis, Foot" OR "Foot Orthotic Devices" OR "Device, Foot Orthotic" OR "Devices, Foot Orthotic" OR "Foot Orthotic Device" OR "Orthotic Device, Foot" OR "Orthotic Devices, Foot" OR "Foot Arch Supports" OR "Arch Support, Foot" OR "Arch Supports, Foot" OR "Foot Arch Support" OR "Support, Foot Arch" OR "Supports, Foot Arch" OR "Orthotic Shoe Inserts" OR "Insert, Orthotic Shoe" OR "Inserts, Orthotic Shoe" OR "Orthotic Shoe Insert" OR "Shoe Insert, Orthotic" OR "Shoe Inserts, Orthotic" OR "Orthotic Insoles" OR "Insole, Orthotic" OR "Insoles, Orthotic" OR "Orthotic Insole") AND TS=("Control Groups" OR "Comparative Study" OR Control OR Comparison OR Treatment OR Intervention) AND TS=("Pain Measurement" OR "Pain Relief" OR "Radiographic Parameters" OR "Foot Pain" OR Improvement)

**Embase:** ('flatfoot'/exp OR 'talipes valgus' OR 'valgus' OR 'talipes' OR 'splayfoot' OR 'flat foot' OR 'foot, flat' OR 'pes planus' OR 'flat feet' OR 'feet, flat' OR 'flatfeet' OR 'vertical talus' OR 'talus, vertical' OR 'rigid flatfoot' OR 'flatfoot, rigid' OR 'convex foot' OR 'convex pes valgus' OR 'pes valgus, convex' OR 'vertical talus, congenital' OR 'congenital vertical talus' OR 'talus, congenital vertical' OR 'rocker-bottom foot' OR 'foot, rocker-bottom' OR 'rocker bottom foot' OR 'pes valgus, congenital convex' OR 'talipes calcaneovalgus' OR 'calcaneovalgus, talipes' OR 'flexible flatfoot' OR 'flatfoot, flexible' OR 'acquired adult flatfoot deformity' OR 'children' OR 'pediatric') AND ('foot orthosis'/exp OR 'foot orthoses' OR 'orthoses, foot' OR 'foot orthosis' OR 'orthosis, foot' OR 'foot orthotic devices' OR 'device, foot orthotic' OR 'devices, foot orthotic' OR 'foot orthotic device' OR 'orthotic device, foot' OR 'orthotic devices, foot' OR 'foot arch supports' OR 'arch support, foot' OR 'arch supports, foot' OR 'foot arch support' OR 'support, foot arch' OR 'supports, foot arch' OR 'orthotic shoe inserts' OR 'insert, orthotic shoe' OR 'inserts, orthotic shoe' OR 'orthotic shoe insert' OR 'shoe insert, orthotic' OR 'shoe inserts, orthotic' OR 'orthotic insoles' OR 'insole, orthotic' OR 'insoles, orthotic' OR 'orthotic insole') AND ('control group'/exp OR 'comparative study'/exp OR 'control' OR 'comparison' OR 'treatment' OR 'intervention') AND ('pain measurement'/exp OR 'pain relief'/exp OR 'radiographic parameters' OR 'pain relief' OR 'foot pain' OR 'improvement')

**Cochrane Library:** (("Flatfoot" OR "Talipes Valgus" OR "Valgus" OR "Talipes" OR "Splayfoot" OR "Flat Foot" OR "Foot, Flat" OR "Pes Planus" OR "Flat Feet" OR "Feet, Flat" OR "Flatfeet" OR "Vertical Talus" OR "Talus, Vertical" OR "Rigid Flatfoot" OR "Flatfoot, Rigid" OR "Convex Foot" OR "Convex Pes Valgus" OR "Pes Valgus, Convex" OR "Vertical Talus, Congenital" OR "Congenital Vertical Talus" OR "Talus, Congenital Vertical" OR "Rocker-Bottom Foot" OR "Foot, Rocker-Bottom" OR "Rocker Bottom Foot" OR "Pes Valgus, Congenital Convex" OR "Talipes Calcaneovalgus" OR "Calcaneovalgus, Talipes" OR "Flexible Flatfoot" OR "Flatfoot, Flexible" OR "Acquired Adult Flatfoot Deformity" OR children OR pediatric) AND ("Foot Orthoses" OR "Orthoses, Foot" OR "Foot Orthosis" OR "Orthosis, Foot" OR "Foot Orthotic Devices" OR "Device, Foot Orthotic" OR "Devices, Foot Orthotic" OR "Foot Orthotic Device" OR "Orthotic Device, Foot" OR "Orthotic Devices, Foot" OR "Foot Arch Supports" OR "Arch Support, Foot" OR "Arch Supports, Foot" OR "Foot Arch Support" OR "Support,
